# Annotations

**Published:** 1899-04-29

**Authors:** 


### Annotations.

### "Successful" or "Efficient" Vaccination.

Perhaps in view of the latitude allowed by the Vaccination Act in regard to being vaccinated at all, some people may think it is hardly worth while talking about measures to ensure that such vaccination as is done shall be efficient. We do not agree with them. Now is exactly the time when, if vaccination is done at all, it should be done in such a manner as to be thoroughly protective. No one can read the evidence given before the Royal Commission without seeing how the whole question was throughout fogged by the fact that a large proportion of the vaccination, although "successful" enough in the sense that it had "taken" as the phrase goes—that is, that at least a vesicle had been produced—was far from "efficient" in the sense insisted on by the Local Government Board, and, in view of the epidemics which are before us, it seems more than ever of importance that there shall be a firm and distinct line between the vaccinated and the unvaccinated. With the object of directing the attention of the Local Government Board to this matter the Jenner Society has forwarded to that body a series of resolutions, urging among other things that the certificate of "successful" primary vaccination should in all cases record the number of punctures or groups of punctures, and the number of separate normal vesicles or groups of vesicles which have been produced, and that a permanent record of the facts so certified should be kept for future reference. They also wish that the certificate should bear on the face of it some statement of what, in the opinion of the Local Government Board, constitutes "efficient" as distinguished from "successful" vaccination, so that when the fond mother carries her baby home in triumph, with its one tiny mark upon its dear little arm, she shall know that all her trouble has been for nothing, and that, although she, with the collusion of the doctor, has been "successful" in evading the spirit of the Act, she has failed in giving her child "efficient" protection against disease. This does not seem unreasonable, and certainly in the event of an epidemic it would be a great advantage to know which are the badly vaccinated ones, who, above all others, should be revaccinated without delay.

### The Salvation Army.

The aspect, to many people a completely new aspect, under which the Salvation Army was presented to the public by the meeting held last week at the Mansion House, recalls one of the most striking passages of Macaulay's famous description of the Puritans. "People," he says, "who saw nothing of the godly but their uncouth visages, and heard nothing from them but their groans and whining hymns, might laugh at them. But those had little reason to laugh who encountered them in the hall of debate or in the field of battle. These fanatics brought to civil and military affairs a coolness of judgment and an immutability of purpose which some writers have thought inconsistent with their religious zeal, but which were in fact the necessary effects of it." In like manner, the inhabitants of streets frequented by the processions of the "Army," with their brass bands and their banners, with their singing and their shouting, may be excused if they think of the performers in no other light than as an unmitigated nuisance, for the suppression of which all the powers of law and of public opinion may properly be invoked. The religious tenets of the processionists, which, so far as we are acquainted with them, are mainly those of the sect commonly described as "Calvinistic Methodists," probably appeal only to a small section of the community. But all this, if we may accept the conclusions of the very shrewd and practical speakers at the Mansion House—of such men as Mr. Cecil Rhodes, Lord Justice Rigby, and the Earl of Aberdeen—is but the froth which mantles upon strong drink. The work of the "Army," in rescuing the waste products of civilisation from the pitfalls by which they have been ensnared, and in restoring them to honesty and to usefulness, has been sufficiently meritorious to induce the Legislatures of fifteen colonies to make and to continue pecuniary grants for its assistance. It has been organised and conducted, as all successful undertakings must be, upon strictly rational and business principles, free alike from fanaticism and from hysteria, and only so far connected with the religious aspects of the Army as to derive from them a motive force sufficient to furnish a multitude of willing workers capable of surmounting difficulties which to most people would have appeared insuperable. Such a result as this rises superior to shibboleths, and establishes the efficacy and the wisdom of the methods by which it has been achieved. Mr. Bramwell Booth stated at the Mansion House that the organisations of the Army in this country were maintained last year at a total cost of £150,000; and that no less than £143,000 of this amount had been furnished (or, as we understood, repaid) by the persons who had derived benefit. If this be so, sensible people may forget the absurd travesty of military words and titles, may even recognise that this travesty itself may have its uses, and may cordially render assistance to an undertaking which, under whatever names or colours, so thoroughly realises the first great principles of human brotherhood, and fulfils the first great requirements of Christian charity.

### The Infinitely Little.

A London parish has put forth a well-intentioned paper on the precautions which should be taken by the public in order to diminish the diffusion of tuberculosis; and has given therein an account of the tubercle bacillus which is distinctly funny, and which the Medical Officer of Health concerned should rectify, for the sake of his own reputation, in any subsequent edition of the document which may be required. Readers are gravely informed that the bacillus in question is so minute that "many millions" of them could "stand on the point of a common sewing needle." This appears to be a plagiarism from the Mahomedan account of certain angels; and it would be better to adopt a more common and certainly more truthful way of assisting persons who are not familiar with microscopic objects to form some approximate notion of the size and shape of a bacillus. This may be done by saying that one million individuals, arranged in a thousand rows of a thousand each, could be placed upon a penny postage stamp. Absurd exaggeration, of whatever kind, is more liable to defeat the ends of its employers than to promote them.

